# An update on regulation of the polymodal TRPV4 channel by protein phosphorylation

**DOI:** 10.1080/19336950.2025.2611698

**Published:** 2026-01-19

**Authors:** Aravind Parthasarathy, David X. Zhang

**Affiliations:** a Department of Medicine, Cardiovascular Research Center, Medical College of Wisconsin, Milwaukee, WI, USA; b Department of Pharmacology and Toxicology, Medical College of Wisconsin, Milwaukee, WI, USA

**Keywords:** Transient receptor potential vanilloid 4 receptors (TRPV4), protein kinases, PKC, PKA, hypotonic cell swelling, arachidonic acid (AA)

## Abstract

TRPV4 is a polymodal Ca^2+^-permeable cation channel activated by diverse stimuli via various pathways and has been one of the difficult membrane proteins to comprehend, like other TRP channels. However, a broad range of functions and pathological conditions associated with these channels continues to fascinate researchers to study them. One of the major regulatory pathways of these channels is through protein phosphorylation catalyzed by various kinases (e.g. PKC, PKA, SGK1, and Src kinase) in a stimulus-specific manner. Several sites of protein phosphorylation have been identified in both N- and C-terminal tails located in the cytosolic region of the channel. One critical phosphorylation-mediated regulatory pathway involves the C-terminal phosphorylation of Ser-824 residue, which has been implicated in activation/sensitization of the channel and its functioning in cells. Due to the lack of structural evidence on the N- and C-terminal tails (largely intrinsically disordered), it remains a challenge to understand the molecular mechanisms involved in their regulation of the TRPV4 channel. However, recent studies have provided new insights into the potential mechanisms of phosphorylation regulation of the channel and helped unravel the complexity of TRPV4 regulation pathways. This review provides an updated summary of the regulatory role of post-translational regulation through phosphorylation, the kinases and residues involved in phosphorylation of the TRPV4 channel. Furthermore, we discuss the importance and potential mechanisms of the C-terminal domain, harboring the Ser-824 residue, in the regulation of channel activation and proper functioning.

## Introduction

Transient receptor potential (TRP) channels, first identified almost five decades ago [1], have been divided into six subfamilies in mammals based on sequence homology in the 2002 nomenclature by Montell et al. [2] and Clapham et al. [3]. They include TRPC (Canonical), TRPM (Melastatin), TRPV (Vanilloid), TRPA (Ankyrin), TRPP (Polycystin), and TRPML (Mucolipin) [4], in addition to several other subfamilies in yeasts and invertebrates [5,6]. TRP channels are expressed in various tissues and cell types. They contribute to a wide range of functionalities, such as mechano-sensation, signal transduction in response to osmo-chemical forces, mitochondrial function, and lysosomal trafficking [7].

TRP channels are often non-selective cation channels, with most of them permeable to Ca^2+^ ions. For example, TRPV1-3 and TRPA1 can pass through Ca^2+^ ions, in addition to other monovalent, divalent, and some larger cations [8]. TRPV4 is also a non-selective cation channel, significantly permeable to Ca^2+^ over other cations [9]. In contrast, TRPV5 and TRPV6 are highly Ca^2+^-selective and have specialized selective filters for Ca^2+^ ions to pass through [10]. TRPM4 and M5 conduct monovalent Na^+^ and K^+^ [11], whereas M3 conducts Ca^2+^ and Mn^2+^ [12]. TRPM7 is permeable to most of the divalent cations and trace metals [13], and TRPM6 is permeable to trace metals, including Ca^2+^ and Mg^2+^ [14]. Further, TRPC (1–6) channels are non-selective cation channels, permeable to mostly Ca^2+^ over other ions.

TRP channels are activated and regulated by diverse mechanisms, involving G protein-coupled receptor (GPCR)-related mechanisms, mechanical stimuli, direct interactions with lipids and other proteins, and post-translational modifications through phosphorylation and ubiquitination [15]. For example, physical stimuli such as changes in osmolarity, mechanical stress, and temperature can open TRP channels by inducing conformational changes in the protein [16,17], while chemical stimuli, including growth factors, can also regulate the gating of these TRP channels [18]. Voltage-dependent regulation of TRP channels has also been studied in TRPV1 channels [19]. TRP channels are also sensitive to membrane lipids such as phospholipid phosphatidylinositol 4,5-bisphosphate (PIP_2_) [20,21] and other lipid mediators [22,23]. Interestingly, activation of TRP channels can be selectively regulated by protein phosphorylation of discrete serine/threonine and tyrosine residues through PKA, PKC, and SRC kinases, as reported in the regulation of arachidonic acid (AA)-induced TRPV4 activation by PKA/PKC-mediated Ser-823 and Ser-824 phosphosrylation [24–26] and by other post-translational modifications like ubiquitination [27], and glycosylation [28].

In this review, we provide an update on studies related to the TRPV4 basic structure, function, and its regulation through post-translational modifications. This review focuses on TRPV4 phosphorylation sites catalyzed by different kinases and their role and potential mechanisms of action in regulating TRPV4 activity in a stimulus-specific manner.

## Expression, function, and structure of the TRPV4 channel

TRPV4 was discovered as a multimodal ion channel activated by diverse physical stimuli, including warm temperature [29], hypotonic cell swelling [30], membrane stretch [31], shear stress [32,33], as well as by low pH [34]. Subsequent studies revealed that TRPV4 is also sensitive to various chemical stimuli, including synthetic agonists like [35] GSK1016790A, 4-alpha-phorbol 12, 13-didecanoate [36] and other phorbol esters [37]. Several endogenous lipid molecules, such as arachidonic acid (AA) and its metabolites [24,25,38], phosphatidylinositol 4,5-bisphosphate (PIP_2_) [39], inositol 1,4,5-trisphosphate (IP_3_) [40], and endocannabinoids [41] activates or regulates the TRPV4 channel. Further, proteins like PACSIN are also known to bind and modulate TRPV4 activity [42,43]. Certain synthesized chemicals like HC-067047 [44] and GSK2798745 [45] are known to inhibit the TRPV4 activity as well.

TRPV4 is expressed in numerous cell types, including vascular endothelial cells, smooth muscle cells, neurons, skin epithelial cells, pancreatic cells, and nephrons [46–49]. One such example is the vascular system, where TRPV4 converts diverse hemodynamic and chemical stimuli into Ca^2+^ signals in endothelial cells and thereby regulates the vascular tone [46]. And in the kidneys, TRPV4 detects osmotic stimuli and regulates blood pressure in the presence of higher salt intake [50]. In the neuronal system, TRPV4 helps in promoting brain development and neuro-regeneration [7]. Recent studies have shown that TRPV4 channels located in organelles like mitochondria contribute to Ca^2+^ buffering and the regulation of mitochondrial metabolism [51–53]. Further, endoplasmic reticulum (ER), an important organelle involved in the folding, assembly, and trafficking of the channel to the plasma membrane may also express TRPV4. TRPV4 expressed on endoplasmic reticulum membrane regulates the ER-mitochondria contact points during Ca^2+^ buffering, and facilitates Ca^2+^ efflux into the cytosol during the IR-induced temperature elevation in spiral ganglion cultures via IP3 and ryanodine receptors [54–56]. This diversity in expression and functionality of TRPV4 makes them an important therapeutic target for different pathological conditions in the vasculature, heart, kidney, and neuronal systems. Presently, over 80 genetic mutations have been found in various TRPV4 channelopathies in humans, including some skeletal and peripheral nervous system disorders [57–59].

TRPV4 is a typical homo-tetrameric channel formed from four identical subunits, with each subunit composed of 871 amino acids, although a few studies have reported hetero-tetrameric channels assembled from subunits of TRPV4 along with those of TRPC1, TRPP2, or TRPV1 channels [60–62]. TRPV4 harbors a transmembrane domain (TMD) consisting of a voltage-sensor-like domain (S1-S4) and a pore domain (S5- P-loop -S6), which are linked through an S4-S5 linker. The TRP helix is a unique structure of TRPV4 and many other TRP channels that is situated below the S1-S4 TMD domain and runs almost parallel to the inner surface of the bilayer membrane. The cytosolic N-terminus consists of a proline-rich domain (PRD) and an ankyrin repeat domain (ARD), and the C-terminus contains a calmodulin binding domain (CBD) and a PDZ-like domain [9,44,63,64]. The distal N- (~150 aa) and C- (~90 aa) termini consist of intrinsically disordered regions (IDR), which are important in regulating the channel activity [65].

TRPV4 contains several conserved regions that are critical for its function and regulation, including N-terminal region (PRD and ARD), transmembrane region (TMD and pore domain), and C-terminal region (TRP box and CBD) [41,65,66]. Notably, two phosphorylation residues are identified in the conserved N-terminal region, i.e. Tyr-110 and Ser-162, and a well-studied Ser-824 residue at the conserved C-terminal region [24,25].

## Post-translational modifications in TRPV4

Post-translational modifications (PTMs) of the TRPV4 channel include phosphorylation, ubiquitination, glycosylation, acetylation, and palmitoylation. These modifications play an important role in regulating channel activity, membrane trafficking, protein-protein interaction, and channel protein assembly. Previous studies have also shown that these PTMs contribute to dysregulation of the TRPV4 channel and thereby lead to various diseases like skeletal dysplasia, neuropathies, and cardiovascular diseases [67,68].

### Phosphorylation in TRPV4 channels

As one of the most conserved mechanisms in protein regulation, phosphorylation in ion channels can regulate the channel gating, ion permeability, sensitization/desensitization, and protein expression and trafficking [28]. In TRPV4 channels, phosphorylation has been extensively studied for its regulatory role in channel activation by different stimuli [41], and in the trafficking and expression of channels [69]. Several protein kinases have been identified in TRPV4 phosphorylation at different residues and consequently in TRPV4 activation in response to different stimuli [24,25,37,70–72], including PKC, PKA, SGK, and Src kinases. Further, one member of Src kinase family (Lyn) has been shown to directly interact with TRPV4 during integrin-dependent activation of the TRPV4 channel. Similarly, TRPV4 interacts and is regulated by other cytoskeletal proteins such as microtubules and actin [73–76]. A more comprehensive review of phosphorylation and kinases is discussed in later sections of the review.

### Ubiquitination of TRPV4 channels

Protein ubiquitination was traditionally associated with protein degradation (endocytosis). Over the years, it has been recognized for a non-canonical role in protein localization, trafficking, and regulatory roles in transmembrane proteins [77,78]. As reported by Wegierski et al., protein ubiquitination regulates the expression of TRPV4 channels at the plasma membrane through channel internalization rather than degradation, resulting in reduced basal activity of TRPV4 heterologous expressed in HEK 293 cells. AIP4 (NEDD family of E3 ubiquitin ligase) played a prominent role in regulating TRPV4 and TRPC4 by promoting endocytosis, subsequently decreasing the overall surface expression (basal activity) of the channels on the cell membrane but not in other TRP channels, indicating a more selective role in the regulation of these TRP channels [27]. Subsequent studies have shown that ubiquitination and functional downregulation of TRPV4 are mediated by β-arrestin 1 in HEK293 cells [79], which acts as an adapter for the AIP4 ligase responsible for TRPV4 ubiquitination. Interestingly, recent studies have shown that ubiquitination of multiple lysine residues by NEDD family of E3 Ub ligases (other than AIP4) in the N-terminal intrinsically disordered regions (Lys-77, 101, 130, 136, and 147) increases the TRPV4 channel activity without affecting the cell surface expression of TRPV4 in HEK 293 cells, whereas the other ubiquitinated sites found in the ARD and C-terminal region of the channel did not affect the TRPV4 channel activity [80]. Thus, enhanced ubiquitination can rescue the gain of channel function characteristic of enhanced channel activity. Further, several other non-functional ubiquitinated sites are curated from the phospho-proteomics data, such as Lys177, 192, 197, 352, and 766 [81], as illustrated in Figure 2.

**Figure 1.**
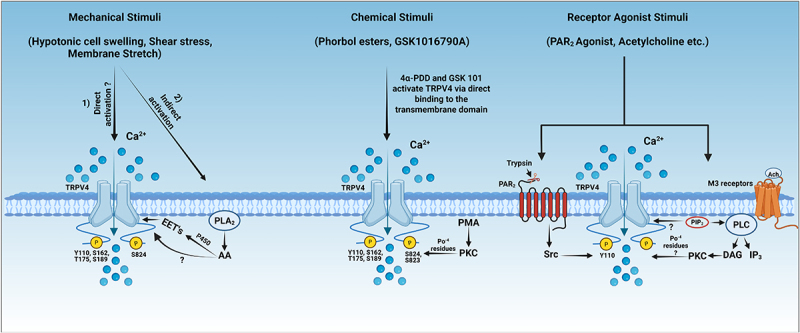
**Proposed activation mechanism of TRPV4 channels by mechanical, chemical, and receptor agonist stimuli and their regulation by phosphorylation.** TRPV4, a polymodal channel activated by diverse stimuli including mechanical, chemical, and receptor agonist stimuli. TRPV4 can be activated directly by mechanical stimuli or indirectly via endogenous lipid mediators, as seen in response to shear stress, hypotonic cell swelling, and membrane stretch. Lipid molecules such as arachidonic acid (AA) and its metabolites (EETs) can directly activate TRPV4 channels, although their mechanisms of action have yet to be determined. Chemical stimuli like phorbol esters (4α-PDD and GSK101) bind to a specific pocket in the transmembrane domain of TRPV4 to open the channel, wherein, Asn-474 residue is found to be an important residue for binding these molecules. Receptor agonist stimuli such as acetylcholine (Ach) binds to M3 receptors to activate PLC, which hydrolyzes PIP_2_ to DAG and IP_3_ and consequently results in a decrease in PIP_2_ and/or an increase in PKC-mediated TRPV4 phosphorylation to activate the TRPV4 channel. Protease-activated receptor-2 (PAR 2), a G-protein coupled receptor regulates TRPV4 channel through tyrosine phosphorylation at Tyr-110 residue by Src kinases. Created in https://biorender.com.

### Glycosylation of TRPV4 channels

Protein glycosylation, the covalent addition of a sugar moiety to a protein macromolecule, can occur through an enzymatic or non-enzymatic process. This glycosylation can be N-linked or O-linked; however, only N-linked glycosylation has been identified in the TRPV4 channel, specifically at the Asn-651 residue adjacent to the pore-forming loop of the channel. A mutation in this region resulted in reduced glycosylation but increased channel activity [82]. This is accompanied by an elevated level of slow-migrating bands on Western blots, indicating a more glycosylated or mature TRPV4 protein in TRPV4-expressing cells. Intriguingly, it has also been shown that mutation of 20 amino acids in the distal C-terminal region of the channel (838–857) results in mostly non-glycosylated TRPV4 protein trapped in the endoplasmic reticulum, which can subsequently promote protein degradation and loss of channel activity [83]. It is currently unclear how this C-terminal region regulates glycosylation, maturation, or trafficking of TRPV4 channels.

### Other PTMs of TRPV4 channels

Protein palmitoylation has been shown to regulate some TRP channels, like TRPM7 [84]. However, there is no direct evidence on this or other PTMs of TRPV4 channels other than an indirect effect, wherein down-regulation of the MAPK pathway inhibits the activation of histone acetylation, resulting in reduced TRPV4 mRNA and protein expression in neuronal cells [85].

## Phosphorylation regulation of the TRPV4 channel with various stimuli

As described above, TRPV4 is a polymodal channel activated by numerous physical and chemical stimuli, including shear stress, cell swelling, lipid mediators like arachidonic acid (AA), epoxyeicosatrienoic acids, endocannabinoids, and phorbol esters. Not surprisingly, extensive studies have been reported regarding some rather complex regulatory mechanisms of TRPV4 based on the stimuli involved in activation of the channel [65,86,87]. The effect of phosphorylation in regulating the activation of these channels by different stimuli is discussed in this section.

### Mechanical stimuli

Activation of TRPV4 by shear stress was first reported by Gao et al. [16] and confirmed by Kohler and colleagues in the early 2000s[88,89]. Ca^2+^ influx through TRPV4 channels induced by shear stress regulates the contractile state, mainly vasodilation of the vessels by producing vasodilating factors in endothelial cells [33,88]. An increase in [Ca^2+^]_i_ concentrations through TRPV4 channels in response to shear stress results in vasodilation, through nitric oxide or EDHF pathways [90,91]. Shear stress may activate TRPV4 directly or through indirect pathways involving lipid mediators such as EET’s etc., as shown in Figure 1. The regulation of these channels during shear stress involves tyrosine phosphorylation at N- and C-terminal residues in the intrinsically disordered regions (IDR) (Tyr-110 and Tyr-805) wherein [71], N-terminal residue Tyr-110 is phosphorylated by the Src family of kinases, and this phosphorylation was further increased by shear stress, subsequently increasing TRPV4 Ca^2+^ responses in endothelial cells [92].

Hypotonic cell swelling produces membrane tension and is another commonly used mechanical stimulus for TRPV4. The increased tension activates TRPV4, allowing Ca^2+^ ions to enter and trigger downstream effects. Initial studies in HEK 293 cells suggested Tyr-253 residue phosphorylation regulates TRPV4 activation during hypotonic swelling [70] but this finding was not reproducible in subsequent studies as the tyrosine kinase inhibitors had no inhibitory effect on the activation of TRPV4, and the hypotonic cell swelling activated TRPV4 with the Y253F mutation [93]. Further studies proposed that shear stress, membrane stretch, and hypotonic cell swelling can activate TRPV4 through PLA_2_-dependent formation of arachidonic acid and its metabolites (epoxyeicosatrienoic acid) via cytochrome P450 epoxygenase-dependent pathway [93]. This mechanical activation of the TRPV4 channel is sensitized by phosphorylation of serine and threonine residues by PKC (at the N-terminal Ser-162, Thr-175, and Ser-189) and PKA kinases (at the C-terminal Ser-824) [37]. However, recent studies have also shown that hypotonic swelling can induce the activation of TRPV4 in the absence of PLA2 or protein kinase activity. For example, in Muller cells of retina and xenopus oocytes, TRPV4 can be activated independent of PLA_2_ and its downstream metabolites, as well as protein kinases [94,95] suggesting that specific mechanisms involved in hypotonicity-induced activation of TRPV4 channels may be cell-type dependent.

By serving as potential endogenous activators of TRPV4 in response to mechanical stimuli, lipid mediators like Epoxyeicosatrienoic acids (EETs), metabolites of arachidonic acid (AA), can activate TRPV4 channels as shown in Figure 1. Previous studies have shown that 5,6-epoxy-eicosatrienoic acid (5,6-EET) can also directly interact with the channel at K535 residue [96].

Currently, a prevailing paradigm is that arachidonic acid is metabolized into EETs to activate TRPV4 channels through the cytochrome P450 pathway. However, in recent years, our group has published experimental evidence of TRPV4 activation by the parent lipid molecule arachidonic acid in endothelial cells. Moreover, we have proposed a hypothetical model for arachidonic acid induced TRPV4 activation, for example, through direct interaction of arachidonic acid with the TRPV4 channel around a putative arachidonic recognition sequence (ARS) to induce channel opening. In addition, arachidonic acid-mediated gating of TRPV4 is tightly regulated by the phosphorylation of C-terminal residues, including Ser-823 and Ser-824 [24,25,38,97].

### Chemical stimuli

Phorbol derivatives have been studied extensively in the activation of TRPV4 channels and their regulation by phosphorylation. 4α-Phorbol 12,13-didecanoate (4α-PDD) and phorbol 12-myristate 13-acetate (PMA) are the phorbol derivatives that can activate TRPV4 channels. Recent structural studies have shown that 4α-PDD directly binds at the base of the S1-S4 bundle. Furthermore, certain important residues were found to be crucial for their binding (Asn-474, Asp-546A, Tyr-591) and activation of the channel [44]. PMA, as a traditional PKC activator, can phosphorylate many of the regulatory serine/threonine residues in TRPV4 (e.g. Ser-162, Thr-175, Ser-189, Ser-823, and Ser-824) [24,25,37]. Although PKC inhibitors largely blocked PMA-induced activation of TRPV4 channels, previous studies speculate that PMA can bind directly and activate the channel independent of PKC pathways [16].

GSK1016790A, a selective potent agonist of TRPV4, binds and activates the channel leading to Ca^2+^ influx in cells. It activates the channel largely independent of the phosphorylation regulatory mechanism, through binding directly to the transmembrane domain of TRPV4 as shown in Figure 1. Recent studies have shown that 4α-PDD, GSK1016790A (agonist), and GSK 2798745 (antagonist) bind within the same cavity, suggesting that these chemical ligands share a few of the interaction sites in the TRPV4 channel [64,98]

### Receptor agonist stimuli

In endothelial cells, the receptor agonist acetylcholine (Ach) induces activation of TRPV4 channels, and this activation also requires PKC-mediated phosphorylation [89,90,99] as shown in Figure 1. For example, Ach has been shown to induce TRPV4-mediated Ca^2+^ influx and subsequent nitric oxide (NO)-dependent vasodilation, which involves a specific form of PKC, i.e. PKCα, in endothelial cells [100]. Protease activated receptor–2 (PAR_2_) agonists can activate TRPV4 to increase [Ca^2+^]_i_ in cells during inflammation. The activation of TRPV4 by PAR_2_ agonists is dependent on Tyr-110 phosphorylation by tyrosine kinases [101]. Activation of PAR_2_ also generates a signal through lipid mediators like arachidonic acid and its metabolites to induce the activation of TRPV4 [98].

## Kinases involved in phosphorylation of TRPV4

**Figure 2.**
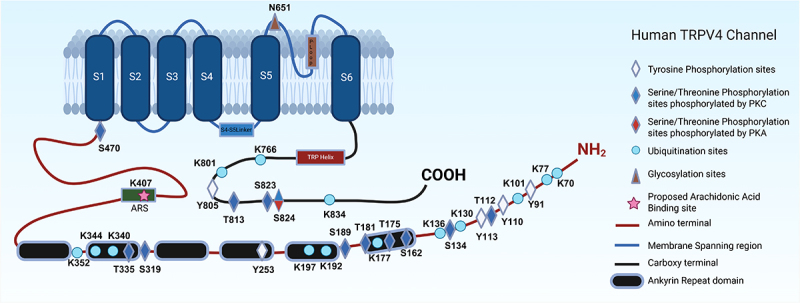
**A diagram of post-translational modification (PTM) sites in the TRPV4 channel.** TRPV4 channel harbors most of the PTM sites in the intracellular domains, except the glycosylation site located in the extracellular pore-loop. The cartoon depicts the currently known PTM sites numbered according to human TRPV4, including sites of phosphorylation catalyzed by different kinases (tyrosine and serine/threonine kinases), ubiquitination, and glycosylation, with each labeled in respective shape/color. Further, one proposed binding site for arachidonic acid in the ARS region (harboring K407 residue) is also shown in the cartoon. Created in https://biorender.com.

Protein kinase A (PKA), Protein Kinase G (PKG), and Protein Kinase C (PKC) family of kinases, tyrosine kinases (Src kinases), Ca^2+^/calmodulin kinase II (CaMKII), and mitogen-activated protein kinase (MAPK) pathways are involved in the activation, regulation, and downstream signaling pathways for TRPV4 channel function. Different phosphorylation sites associated with corresponding kinases are shown in Figure 2.

### Protein kinase C

PKC phosphorylates serine/threonine residues and is activated by Ca^2+^ and diacylglycerol (DAG). PKC isoforms differ in their ability to activate the pathway with Ca^2+^ and DAG alone or in combination with both inducers [102]. The PKC pathway is found to be predominant in the activation/regulation of the TRPV4 channel. Several S/T residues are phosphorylated by PKC activators like PMA in the N- and C-terminal of the channel with various stimuli, indicating the importance of PKC in the regulation of TRPV4 [16,25,37,100,103–105]. Most of the target sites for PKC are in the cytoplasmic domain, along the tails of N- and C-termini (Ser-162, Thr-175, Ser-189, Ser-823, and Ser-824) according to the previous studies.

### Protein kinase A

PKA phosphorylates many target proteins and is activated by the cyclic AMP secondary messenger generated in response to extracellular signals. In TRPV4, they catalyze the phosphorylation of serine/threonine residues, especially Ser-824, during hypotonic swelling, hydrogen peroxide (H_2_O_2_), and arachidonic acid stimuli. TRPV4 activation by arachidonic acid is primarily regulated through the PKA pathway at Ser-824 and less so at Ser-823 residues. Co-expression of the adaptor protein AKAP79 with PKA and PKC enhances the sensitization of TRPV4 [24,25,37,38]. Furthermore, PKA-mediated TRPV4 phosphorylation regulates the activity of TRPV4 channels in endothelial cells during acute hypoxic exercise [106].

### Src kinase

Src family of kinases regulates TRPV4 through tyrosine phosphorylation and is a non-receptor kinase. They phosphorylate certain residues (Tyr-110, Tyr-805, and Tyr-253) in the channel to activate the downstream signaling cascades [70,71,107]. Src kinases interact with TRPV4 and have implications in mechanical hyperalgesia, diabetes, cell volume regulation, hypotonicity, and inflammation [75,107–109]. However, further studies are needed regarding the main phosphorylation sites of Src kinases in the TRPV4. Mass spectrometry studies have revealed that Tyr-110 and Tyr-805 are phosphorylated through tyrosine kinase; however, the functional role of Tyr-805 is yet to be determined. Through mutational studies, Tyr-253 was found to play a role in hypotonic-induced stress, which is subsequently contradicted by the mass spectrometric studies [71]. The phosphorylation of Tyr-253 was not detectable in HEK293T and MDCK cells in the MS spectra, and the hypotonic cell swelling activated TRPV4 with the Y253F mutant studies as discussed in earlier Mechanical stimuli [93].

### CaMKII

In general, CaMKII is activated through the Ca^2+^ and calmodulin pathway in cells. TRPV4, a Ca^2+^-permeable channel, mediates Ca^2+^ influx leading to CaMKII phosphorylation, resulting in diverse regulatory roles in nervous, cardiac, and vascular systems [110–112]; however, it is unclear whether CaMKII also phosphorylates TRPV4. The interaction of TRPV4 and CaMKII has a major role in cardiac hypertrophy, due to activation of TRPV4 leading to upregulation of Ca^2+^/CaMKII mediated activation of the NFkB-NLRP3 pathway [111]. In the nervous system, elevated intracellular Ca^2+^ by TRPV4 through Ca^2+^/CaMKII-mediated mechanisms disrupted mitochondrial transportation, leading to axonal degeneration in neuropathy [73]. This interaction between TRPV4 and CaMKII is also reported in several carcinomas [113].

## N-terminal regulation by phosphorylation and other factors

Previous studies have shown that the N-terminal domain of TRPV4 is responsible for the sensitivity during osmotic changes, mechanical stimuli, and changes in cell volume [50,86,94,114,115]. Phosphatidylinositol-4,5-bisphosphate (PIP_2_) binding at N-termini IDR in the proline-rich domain and its interaction with other proteins, such as PACSIN, are the major regulatory pathways proposed in sensitization/desensitization in response to osmotic stimuli, thermal stimuli, and lipid binding. Several sites have been proposed for the binding of PIP2 in N-termini and in the ARD regions, yet to be confirmed with structural studies [39,42,43,116,117] because they are different from other subfamilies of TRPV channels, including TRPV1 [118,119] (a close homology of TRPV4), TRPV2 [120], TRPV3 [121,122], and in other Ca^2+^-selective TRPV family channels like TRPV5 [123] and TRPV6 [124].

As described previously, several phosphorylation sites are proposed in the N-terminal domain of TRPV4, which are phosphorylated through PKC and Src kinases in response to different stimuli compared to other TRPV channels like TRPV1, TRPV2, and TRPV3 [125–127]. Major regulatory residues involved in the regulation of hypotonic cell swelling are Ser-162, Thr-175, and Ser-189 by PKC [37]. Mass spectrometric studies have confirmed the phosphorylation status of Tyr-110 and Tyr-253 residues proposed to be involved in shear stress-induced phosphorylation through Src kinases [70,71], along with other residues identified in large-scale mass spectrometry studies (Tyr-91, Thr-112, Tyr-113, Ser-134, Thr-181, Ser-319, Thr-335) as shown in Figure 2. It is to be noted that other TRPV channels possess similar but not homologous regulatory sites in the N-terminal region of the channels, such as Ser-116, Thr-144, and Thr-370 residues phosphorylated by PKA in TRPV1 [128,129], Tyrosine residues 335, 471, and 525 phosphorylated by JAK1 in TRPV2 [127], Thr-264 phosphorylation by ERK in TRPV3 [126], Ser-144 and Ser-299 phosphorylation by PKC in TRPV5 [130], and Ser-184 phosphorylation by PKC in TRPV6 [131].

### Tyr-110

One of the significant and well-studied residues involved in the sensitization/regulation of TRPV4 channel in the N- termini IDR region is Tyr-110. Its phosphorylation by Src family of kinases was first proposed by Wegierski et al., in 2009 with MS spectra and phosphorylation studies. Initial studies suggested Tyr-110 as a modulator of the channel activity with varied stimuli, including heat, mechanical stress, and hypotonic cell swelling [71]. Further, Protease-activated receptor-2 (PAR 2), a receptor involved in the neurogenic inflammation mechanism regulates TRPV4 channel through tyrosine phosphorylation at Tyr-110 residue [101]. Other than Src kinase, PMA (PKC activator) can also indirectly induce the phosphorylation of the Tyr-110 residue, wherein, mutating the Tyr-110 to phenylalanine reduced the Ca^2+^ currents in response to PMA significantly in HeLa cells. Few studies favor the hypothesis that Tyr-110 phosphorylation triggers confirmational changes in TRPV4 affecting its interactions with certain proteins like PACSIN, which binds in the proline-rich domain of the channel. The interaction of TRPV4 with PACSIN inhibits the responses to heat and hypotonic cell swelling but not to phorbol ester 4α-PDD indicating a competitive binding between PACSIN and phosphorylated Tyr-110 in TRPV4 [43,71,117]. Together, these studies highlight the importance of Tyr-110 residue in channel sensitization/regulation with different stimuli, however, a considerable number of studies involving the Tyr-110 residue are yet to be done regarding the exact mechanism of Tyr-110 phosphorylation involved in its regulation of the TRPV4 channel.

## C-terminal phosphorylation sites and their regulatory role

Although not fully resolved by recent cryo-EM structures, the C-termini domain of the TRPV4 channel has been studied extensively for its role in various aspects of channel function and regulation, such as regulation of channel activation and gating mechanisms, protein expression of the channel, trafficking, remodeling of cholesterol dynamics on plasma membrane leaflets, and plasma membrane localization [24,25,37,38,44,71,72,83,132–137]. In terms of channel regulation by protein phosphorylation, recent studies from our group have found a new phosphorylation residue, Ser-823 (a preferential PKC site), in addition to the well-studied residue Ser-824, in the activation of TRPV4 channels by arachidonic acid. Tyr-805 is another phosphorylation site in the C-termini detected by mass spectrometry, and phospho-Thr-813 was identified in adenocarcinoma [138]. PKA, PKC, and SGK1 kinases have been found to phosphorylate these residues in the C-termini.

Similarly, the C-termini domain of other TRPV channels possesses major regulatory phosphorylation sites involved in channel sensitization, calcium binding, and many important downstream processes, including Ser-502, Thr-705, and Ser-801 sites in TRPV1 channels phosphorylated by PKA/PKC/CaMKII [139,140], Ser-760 in TRPV2 phosphorylated by ERK [141], Thr-709 in TRPV5 channels phosphorylated by PKA [142], and Thr-728 in TRPV6 phosphorylated by PKC [131].

### Ser-824

Another significant and most-studied residue involved in activation/regulation of the TRPV4 channel is the Ser-824 harbored in the calmodulin binding domain (CBD) of the C-terminal tail. This Ser-824 residue is conserved in mammals and some vertebrates. Various stimuli like arachidonic acid and its derivatives, hypotonic cell swelling, and H_2_O_2_ induce channel protein phosphorylation at the Ser-824 residue [24,37,143], emphasizing their importance in the regulation/activation of the TRPV4 channel. Several kinases act upon the Ser-824 residues depending on specific stimuli: for example, both PKA and PKC can phosphorylate Ser-824 in response to stimulation by arachidonic acid, but PKA predominates as indicated by PKA-specific inhibitors in our previous studies [24,25,38]. Furthermore, we established that Ser-824 and Ser-823 residues are involved in the activation of TRPV4 by the lipid mediator arachidonic acid. Interestingly, PKA phosphorylation of Ser-824 is seen in hypotonic cell swelling stimuli [37] and SGK1 kinase phosphorylates Ser-824 specifically in heat/4α-PDD [72] stimulus.

Once phosphorylated, Ser-824 can associate with phosphoglucomutase-1 (PGM1) to modulate glucose metabolism [133] and interacts with stromal interaction molecule 1(STIM1) for localizing TRPV4 to the plasma membrane from ER [132]. Further, F-actin interacts with the phosphorylated Ser-824 residue for its calcium influx and to expand cell surface area [144]. Together, these studies highlight the importance of the C-terminal Ser-824 residue in the activation/regulation of TRPV4 channels. Accordingly, structural models involving C-terminal regulation of the channels would provide a mechanistic framework for understanding the activity/regulation/sensitization of the TRPV4 channel, and one such model is proposed by our group when studying arachidonic acid-induced TRPV4 activation and its regulation by phosphorylation of distal C-terminal residues (Ser-824 and Ser-823) [24,25].

### A proposed model for the activation of the TRPV4 channel in response to arachidonic acid or metabolites and its regulation by C-terminal phosphorylation

Previous homology models and recent structural studies of TRPV4 suggest a lipid-binding site for 5,6-EET and phorbol esters near the TRP and pre-S1 helices [24,44,96]. Based on molecular dynamics simulations, previous studies proposed an EET-binding pocket for the activation of TRPV4 channel involving S2-S3 linker (K535, F549, and Q55), S4 (Y591) and S4-S5 linker (R594) that overlaps with predicted 4α-PDD site in S3-S4 sites. We proposed a similar binding site (a hydrophobic crevice) for arachidonic acid between the TRP helix and the putative ARS region (Lys-407) in TRPV4 [41,97] which also involves the C-terminal regulation by the phosphorylation of Ser-823 and Ser-824. According to our model, binding of arachidonic acid expands the hydrophobic crevice under the TRP helix, allowing the outward motion of the TRP helix and activation of the channel. This expansion of the crevice can be restrained if the C-terminal domain is lodged to one or another side of the crevice; however, phosphorylation of Ser-824 and Ser-823 residues may prevent the C-terminal domain docking, thus enabling the expansion of the hydrophobic crevice and sensitizing TRPV4 to activation by arachidonic acid.

In the docked position, Ser-823 and Ser-824 are not easily accessible for relatively bulky kinases like PKA and PKC; therefore, it is likely that there is some kind of quick dynamic equilibrium with partial undocking of the C-terminal helix, long enough for the interaction of kinases with residues to be phosphorylated, but not long enough for the complete process of TRPV4 activation by arachidonic acid. However, this hypothesis requires further testing, and the structure and position of the C-terminal domain need to be further modeled until it is experimentally resolved in a structure of TRPV4 channels.

## Conclusion

Membrane proteins such as TRPV4 channels are the major targets of phosphorylation, involved in the activation/regulation of the channel. TRPV4 is among those important channels involved in various cellular activities across the nervous and cardiovascular systems. Several kinases discussed in the review phosphorylate at distinct sites and induce distinct pathways to regulate the channel activity in response to various stimuli. The effect of phosphorylation involves channel gating, membrane expression, and trafficking.

The largely unresolved N- and C-termini of the TRPV4 channel bear the important regulatory phosphorylation residues involved in the activation/regulation of the channel. Phosphorylation regulation via the N- and C-terminal tail of the channel has been well established, with experimental evidence across different cellular models suggesting an important role in the regulation/activation of the channels with varying stimuli. Ser-824 has emerged as a critical residue phosphorylated by different kinases under varied experimental conditions, leading to enhanced channel opening and the interaction of phosphorylated Ser-824 with other residues/proteins that subsequently activate various metabolic pathways in cells. Given their important roles in the proper functioning of the channel, further structure-function studies of the N- and C-terminal domains may be an effective approach in understanding the activation/regulation of the TRPV4 channel.

## Data Availability

Data sharing is not applicable to this article as no new data were created or analyzed in this study.
